# Remediation: Fe-TAML Takes On Estrogens in Effluent

**Published:** 2008-04

**Authors:** Harvey Black

For more than a decade, endocrine-disrupting chemicals (EDCs) have been an environmental and health worry, linked to cancer and reproductive abnormalities in humans and animals. The European Union’s new REACH (Registration, Evaluation, Authorisation and Restriction of Chemicals) legislation labels them “substances of very high concern,” meaning they will need special authorization to be marketed in Europe. According to a report in the 15 February 2008 issue of *Environmental Science & Technology*, a recently developed chemical agent known as Fe-TAML^®^ may help with one aspect of EDC concerns: it can destroy certain EDCs in wastewater in less than 15 minutes.

EDCs, including various chlorinated organics, plastic additives, and estrogenic compounds, can be found in pesticides, in contraceptives, and in growth hormones given to livestock. These compounds are then excreted in human and animal waste. Existing techniques to degrade EDCs include chlorination and ozonation, but chlorination can yield harmful disinfectant by-products, and ozonation requires expensive equipment.

Although wastewater treatment plants may remove 95% of EDCs, residual levels can persist and affect the environment, says study coauthor Nancy Shappell, a research physiologist with the Agricultural Research Service. Of particular concern, says Shappell, is 17α-ethinyl-estradiol (EE_2_), a synthetic estrogen used in women’s birth control, which can alter reproduction at very small doses.

In this study, Fe-TAML (short for iron–tetraamidomacrocyclic ligand) was combined with hydrogen peroxide and added to sewage effluent in a laboratory setting. The compound successfully destroyed several estrogenic compounds, including EE_2_. Estradiol, the chief endogenous estrogen, was reduced by about 98%. Furthermore, the Fe-TAML “estrogen breakdown products had little to no estrogenic activity,” the researchers write. The mechanism by which the catalyst works was described in detail in the Environews article “Fe-TAML: Catalyst for Cleanup” [*EHP* 114:A656–A659 (2006)].

The treatment could be used to remove EDCs from sewage treatment discharges, says principal investigator Terrence Collins, a professor of chemistry at Carnegie Mellon University and lead inventor of the Fe-TAML catalyst. He suggests the catalytic technique be used as a final treatment for effluent after solids are removed.

The research, says Trevor Stuthridge, a scientist with the New Zealand biomedical research institute Scion Research who has collaborated on Fe-TAML studies, shows that the agent may offer performance advantages over current processes: “This enhanced efficacy means that waste treatment operators have a potential new weapon in the fight to ensure that final discharges from these systems are safer for the environment.”

Collins says that preliminary results showed that no toxic products resulted from using Fe-TAML/peroxide. And he notes that Fe-TAML catalysts have passed a series of aquatic toxicity assays, as reported in the 12 April 2002 issue of *Science*, among other sources. But he cautions that more toxicity studies need to be done to ensure that Fe-TAML does no harm if used to treat drinking water.

Although 1 kg of the catalyst can treat 20,000 tons of water, a large city plant can process many times that amount of water each day. Consequently, says Collins, any chemical treatment “must be very, very cheap for massive-scale applications.” He and his colleagues are currently working to make Fe-TAML competitive on the marketplace.

